# Bayesian approach for localizing cardiac sources in Magnetocardiography using Vectorcardiography based total variational priors

**DOI:** 10.1038/s41598-025-09466-1

**Published:** 2025-07-11

**Authors:** Vikas R. Bhat, Karunakar Kotegar, H. Anitha

**Affiliations:** 1 Department of Biomedical Engineering, Manipal Institute of Technology, Manipal Academy of Higher Education, Manipal, Karnataka 576104 India; 2 Department of Data Science and Computer Applications, Manipal Institute of Technology, Manipal Academy of Higher Education, Manipal, Karnataka 576104 India; 3 Department of Computer Science and Engineering, Manipal Institute of Technology, Manipal Academy of Higher Education, Manipal, Karnataka 576104 India

**Keywords:** Bayesian statistics, Forward problem, Inverse problem, Magnetocardiography, Total variation, Vectorcardiography, Computational biology and bioinformatics, Biomedical engineering

## Abstract

The human heart produces electrical signals to contract and relax its muscles that helps in its blood pumping activities. These electrical impulses give rise to electric potentials on the body surface and tiny magnetic field around the thorax. These functional activities can be investigated using Electro/Magnetocardiogram (E/MCG). The more challenging task in the E/MCG research is to image the cardiac dysfunctions in three dimensions not at the surface level but at the source level and this is called the inverse problem. To solve this, one has to model a generic structure of the discretised heart enclosed in the thorax mesh and their spatial relation with location of the MCG detectors, called forward problem. In this current research, sources in a homogeneous volume conductor model is used in the construction of forward problem. A novel algorithm is implemented that uses Vectocardiography (VCG) signals in the forward problem of MCG. Another objective of this paper includes the utilization of dynamic lead field based on VCG orientations in the inverse problem. In this study, the ill-posed problems are solved using Bayesian approach and the results are compared with the deterministic approach for measurements on noise signals. The analysis revealed that the proposed algorithms with VCG priors (that are extracted from the VCG signals) in the Bayesian framework significantly improved MCG source localization in cases of Myocardial Ischemia. Analysis from the study showed that the proposed algorithms with VCG priors in probabilistic methods significantly captured a good region of spread of the reconstructed borders of inverse solutions. The average spread for deterministic methods was around 3.5 - 4.3cm in the diseased cases with simulated true ruptured region of spread being 2.5cm. In contrast, Bayesian methods and total variation methods with VCG priors reduced the spread to 2.9 to 3.03cm, respectively. The introduction of VCG signals in the forward problem of MCG not only increases the accuracy of cardiomagnetic imaging but also provides a path for more reliable diagnostic tools in cardiology.

## Introduction

Electrocardiogram (ECG) and Magnetocardiogram (MCG) typically measure potentials and magnetic fields, respectively, to reflect the electrophysiological changes that indicate how well the heart is functioning. Heart malfunction is demonstrated by the variations from the typical functional waves impressed or recorded in E/MCG detectors. Despite its limitations in diagnosing cardiac function, ECG is not able to accurately locate problems. Clinicians must either do invasive examinations or gather structural data in order to evaluate such irregularities.Since MCG is a non-contact method and is not impacted by skin conductivity profiles, it has clear benefits over ECG. MCG is highly sensitive to tangentially flowing currents along the thorax, which directly relate to the heart’s electrical activity, whereas ECG measures both radial and tangential currents, with the radial currents influenced by the skin and underlying tissues. As a result, MCG can be considered more accurate for localization than ECG. Additionally, the artefacts that may arise from skin-electrode contact in ECG measurements are avoided in the case of MCG. Many researchers have contributed their work to the non-invasive use of bioelectric fields for localization of cardiac sources. In the biomedical community, a new method called cardio-magnetic imaging has emerged that promises clinicians to evaluate several heart-related conditions without the need for invasive treatments. The reconstruction of the heart waves from E/MCG measurements is called as inverse problem which is ill-posed. The primary idea is to record the measurements of the heart and search for activities that created it in an inverse manner. This can be accomplished by combining the structural and functional knowledge. A few requirements must be built in order to find the inverse solution. The first step is to figure out a forward problem that outlines the anatomy of the heart and its presumed functions. Here, assumed heart surface activities are considered as prior sources. In the literature, there are several methods/models that may be presumed to generate cardiac source activations^1,2^. A transfer matrix connecting the sources and detectors’ spatial coordinates is created for the forward problem. With the help of recorded signals and a spatial matrix that connects the locations of the sources and detectors, the MCG inverse problem reconstructs the cardiac source distribution. However, the biomagnetic inverse problems are ill-posed because of the skin conductivities and geometrical attenuation between the sources and the detector locations; even minor inaccuracies in the recorded data can cause significant variations in the estimated source activity. The common technique used to overcome this type of ill-posed problems is to solve for deterministic approach called as regularization [1], [3]. This method provides a solution constraint of a-priori sources weighted by parameter and a minimum misfit error between the measured and test data [4]. Bayesian linear regression is an alternate approach that is capable of solving the cardiac inverse problems referred in the literature [5],[6]. There are several methods to solve the forward and the inverse problems of MCG: Hämäläinen^3^, J.Sarvas^4^, Mariyappa at al.,^5^ who used analytical approaches. The authors^6,7^, used the invasively recorded epicardial potentials as the prior heart sources and used Tikhonov regularization to estimate the heart activities from the recorded MCG data. Wach and Tilg^1^, Huiskamp et al^2^ modelled the action potentials as prior sources and the MCG signals from this equivalent dipole layer. The cardiac sources were localized using $L_2$ norm with Tikhonov regularization^8^. The above mentioned literature used $L_2$ norm based inverse methods that effectively solves or estimates the source activities of the heart. However, the inverse weight matrices obtained from $L_2$ norm lacked its ability to produce sharper solutions. The authors^9^ proposed a technique called Total variation ($L_1$ norm) based regularization to reconstruct the epicardial potentials. Also, they applied this method to resolve Wolf-Parkinson White syndrome and showed that $L_1$ norm method detected pathways which were not localized by $L_2$ regularization. Their experiments of $L_1$ norm method detected epicardial pacing sites more accurate than the $L_2$ norm.

J.C.Font et al.,^10^ implemented total variation inverse algorithms that discretizes the heart and the torso geometries and solves for heart transmembrane potentials. They developed this algorithm in SCIRun software ^11^and showed that their technique produced more non-smoother results.

$L_1$ and $L_2$ norm regularization techniques are effective approaches to solve an inverse problem by minimizing the misfit between the measured and the output model. L. Zhukov et al.,^12^ considered the spatio-temporal method to localize the EEG sources. They estimated the location and activities of the current sources within the brain from the EEG recordings. The forward model of the brain was constructed with finite element meshes and the potentials were driven on to the scalp and electrodes by solving the linear system. The inverse problems were solved using a non-linear optimization technique of finding the least squares fit. To perform this minimization, the authors used the multi-start downhill simplex search to locate the dipole within a single element of finite element mesh. The simplex search was executed for several trials in order to find the minimum. Another illustration on solving the brain localization using the simplex search technique was presented by D. Weinstein et al.,^13^. In their research work, the authors constructed the lead fields for the spherical models using the nodes and the element basis functions in the forward problem and approximated the brain sources using the simplex optimization technique. R. Van Uitert et al.,^14^ solved the forward and inverse simulations of the brain sources using the MEG data. The authors first simulated the problems on the finite element spherical meshes and applied on the realistic models. They compared the simulation results between the computations containing the volume currents and without the volume currents. In^15^, authors compared the effectiveness of different low resolution minimum norm techniques in localizing brain sources using EEG data, highlighting their performance in terms of localization accuracy and computational efficiency.

As quoted in^16^, the regularization theory gives satisfactory results, but deals less with inaccuracies and uncertainties on models, one can fullfull such problems using a probailistic approach. The authors suggested that, in order to deal with uncertainties on the model when real world situations were faced, probability theory has been invented to handle such notions.

They addressed that the main limitations of the regularization theory is the lack of tools for selecting the regularization parameter.

So, determining the balanced parameter is still an open problem, whereas probabilistic based approach try to overcome such drawbacks. The uncertainties on the known variables (measurement data) are only considered.

They addressed that the Bayesian approach seemed to be the best one that takes into account of both the data inaccuracies and the uncertainty factors in the solution’s prior knowledge (thus providing a good solution to the ill-posed problems). According to the authors, the measures of confidence lacked in the regularization theory could be addressed by Bayesian theory.

Researchers employed ellipsoidal convex models to effectively quantify uncertainty boundaries in measured responses, even when only limited samples are available^17^.

D. Mackay^18^ proposed Bayesian approach to regularization. The regularizing constants were estimated by maximizing the evidence (prior distributions). They showed that the main advantage of Bayesian approach approximated the unknown variables from the known parameters of the observed data with the help of prior knowledge; unlike trial and error method of selecting the regularizer in deterministic approaches.C.M.Bishop^19^ explained and discussed the Bayesian approximations for Neural networks. In this, the author used Gaussian models for both prior and likelihood distributions. Thorsteinn^20^, in his thesis, presented various types of Bayesian approaches to solve the inverse problem of EEG. The author assumed the Gaussian type models to design the hyper-prior distributions. J.J.France^21^ studied Bayesian approach to quantify difference in $L_2$ norm solutions arised from conductivity and mesh discretization. they explained that, eventhough the $L_2$ norm solution provided a satisfactory deterministic results; Bayesian would not only estimates an equivalent maximum a-posteriori (MAP), but also capable of providing a distribution which can be evaluated to study the sources of uncertainty. They used Gaussian priors to model the prior source activities.

However, the Gaussian prior distribution in probabilistic approach yields $L_2$ norm solutions, which results in smooth solutions. In order to obtain sharp results, $L_1$ norm regularizer could be employed in deterministic approach. In contrary to that, Laplacian priors are used in the Bayesian approach to obtain sparse solutions. Babacan et al.,^22,23^ proposed the components of compressive sensing problem using the Bayesian framework. The authors utilized a hierarchical form of the Laplacian prior to generate a sparse model in the unknown signal. They claimed that their proposed study provided the estimate of the uncertainty of the reconstructions.

J.Lee et al.,^24^ applied the total variation prior for identifying the discrete geologic inverse problems. J.M.Bardsley^25^ performed a theoretical analysis of the Laplacian priors in Laplacian increment model to deblur the images. The authors^26^ used Bayesian hierarchical model to deblur images using Total variation (TV) regularization priors. They used this prior and developed an iterative algorithm to deblur the images. Babacan et al.,^27,28^ proposed novel algorithms for TV based image restoration using Variational approximations. The proposed algorithms showed that the posterior distributions were approximated effectively without any assumptions about the hyperparameters and clearly outperformed the existing methods.

So far, however, the literature study of Bayesian approach using TV prior limited its applications in the image processing techniques, there is a motive to study the proposed usage of Laplacian and TV priors in various applications. In the paper^29^, the authors proposed and applied the TV priors in localizing the EEG sources non-invasively. The group applied the similar method in image processing for deblurring and edge detecting applications. In this paper^29^, they used the variational methods to approximate the posterior distributions of the brain sources. They extended their work by testing and validating the simulations on synthetic data. The study of localization of cardiac electrical activity using the MCG measurements provides a great tool in evaluating the localization of cardiac diseases. The diagnosis of complex cardiac diseases such as arrhythmia, myocardial infarctions can be interpreted much better if the use of biophysical and computational heart models were simulated for the analysis. Therefore, the computational imaging of heart is a challenging and promising field of research which might help the clinicians and researchers to analyze the patient-specific heart models. Accordingly, the current research work is challenging in terms of estimating the heart sources in terms of epicardial potential distributions non-invasively and diagnose the diseases more accurately.

In^30^, the authors developed a novel approach by solvin the MCG inverse problem using Bayesian approach with varying spatial matrix updates derived from Vectorcardiography (VCG) signals. The work included localization of the cardiac sources along with the reconstruction of epicardials. The methods presented were less focused on dynamically updating the VCG signals with respect to time and the sparsity was less explored in the source reconstruction.

The objective of this paper is to i. Construct the forward problem of heart by modified transfer matrix built using VCG, ii. Solve the inverse problem by reconstructing the cardiac sources from MCG using proposed method variants - Bayesian inference with Gaussian and total variation prior models with adaptive VCG update in diseased cases.

### Methodology

The first step is to model the heart sources and construct a spatial sensitivity between the sources and the detectors. Source model is defined as the prior assumption of the cardiac activities for a generic functional information about the myocardium. This work utilizes the forward model described in^31^.

Three different forms of Myocardial Infarctions (MI) are simulated: increased R-peak, ST elevated MI, and increased T-peak MI. The transmembrane potentials (tmp) wave’s magnitude and duration were adjusted to mimic these abnormal cases in ECGSIM. i. To obtain a higher R peak, the tmp wave’s rate of ascent was slowed down; ii. To obtain a ST elevated MI, the tmp wave’s magnitude was lowered to 40% without altering its duration. and, iii. To mimic an elevated T peak, the tmp time was decreased. The inverse problem makes use of the spatial parameters that are saved from these sources.

**Fig. 1:**
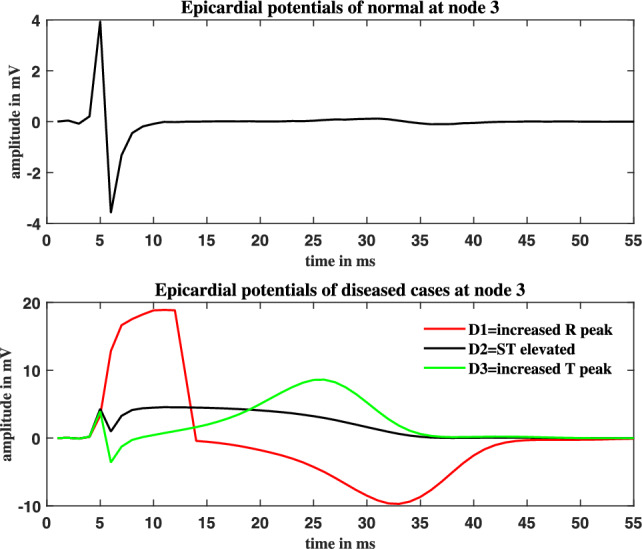
Normal epicardial potentials along with simulated abnormal epicardial potentials at node index 3: Increased R peak in the epicardial wave (D1), elevated ST (D2) and (d) increased T peak (D3).

Around node 3, the infarcted area extended to a distance of 2.5 cm. This range was chosen in order to spread the infarcted activity among the nearby cells in the anterior walls of the heart. The amplitudes of the simulated abnormal epicardial potentials are as shown in the Fig. 1 (second subplot), while the normal epicardial potentials are shown in Fig. 1(first subplot). The amplitude range of epicardial potentials for the normal case was $8mV_{pp}$. The modified magnitude of R peaked case was changed to the range from -10mV to 19mV. The elevated ST case showed no changes in the R peak amplitudes but the ST peaks of epicardial potentials showed an increase of 5mV upstroke from the normal epicardials. The simulated third case (increased T wave) showed 6mV rise in the amplitude of T wave of epicardial potentials.

**Fig. 2:**
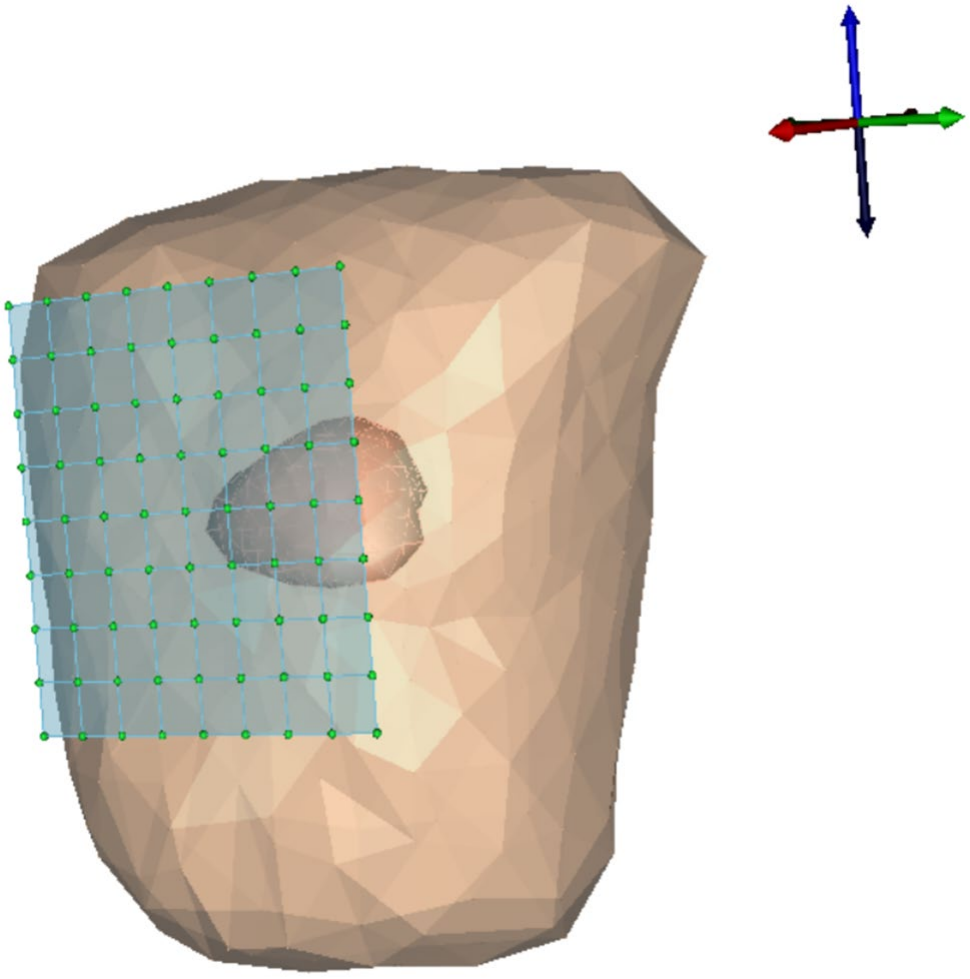
Thorax mesh and heart surface aligned with the MCG detectors.

### Forward problem

After setting up the geometries of the sources (heart) and the sensors (MCG) as shown in Fig. 2, the relation between the *q* sources and the *M* detectors are constructed known as transfer matrix. Fig. 2 shows a $9 \times 9$ array of detectors with an inter-spacing distance of 30 mm. The geometry of detector points was adapted from^32,33^. The standard mathematical formulation of homogeneous forward field matrix^3,4,34^ is given by,

1 $$\begin{aligned} {\textbf{B}}({\textbf{r}}_M, t) = \frac{\mu _0}{4\pi } \int _V P({\textbf{r}}_q, t) \times \frac{{\textbf{r}}_M - {\textbf{r}}_q}{\Vert {\textbf{r}}_M - {\textbf{r}}_q\Vert ^3} \, dV, \end{aligned}$$

where ${\textbf{B}}({\textbf{r}}_M)$ represents the magnetic field at the detector locations ${\textbf{r}}_M$, generated by the epicardial sources which are distributed in the discretized volume space. $P({\textbf{r}}_q, t)$ denotes the strengths of source activities at specific locations ${\textbf{r}}_q$ on the heart.

The conventional lead field is implemented analytically by introducing a unit current dipole on the heart nodes and activating it each time [12], [18]. The formulation of lead field is achieved by:

2 $$\begin{aligned} {\textbf{L}}({\textbf{r}}_M, {\textbf{r}}_q) = \frac{\mu _0}{4\pi } \int \frac{{\textbf{r}}_M - {\textbf{r}}_q}{\Vert {\textbf{r}}_M - {\textbf{r}}_q\Vert ^3} \times P_0. \hat{{\textbf{a}}}_q \, d{\textbf{v}}' \end{aligned}$$

The computational steps to solve the forward problem are given as follows:

1. Load the heart and the torso geometries spatially aligned with the MCG detectors.
2. Insert/place a dipole at the ${{\textbf {r}}}_q$ location ($q = 1$), with strength $P_0 = 1$ in the direction pointed to the unit vector $\hat{{\textbf{a}}}_q = \frac{{{\textbf {r}}}_q}{|{{\textbf {r}}}_q|}$.
3. Compute the field distributions *L* due to the position of the dipole at ${{\textbf {r}}}_1$.
4. Shift the location of the dipole to the adjacent heart node $\delta ({{\textbf {r}}}- {{\textbf {r}}}_q)$ and append the distributed fields $M \times q$ generated from the shifted dipole.
5. Similarly, record the corresponding lead field matrices *L* due to the *q* dipoles covering the entire myocardial nodes.

#### VCG based transfer matrix

The dipole orientations play a major role in the E/MCG inverse problems which represent the depolarization and the repolarization stages of the cardiac muscles. The lead field discussed in the previous section, assumed the prior sources to be fixed and oriented as unit vectors of the locations of the sources with the unit strength^35^. In order to compute forward field, a proposed transfer matrix is constructed based on varying VCG vectors. The extracted coefficients of the transform matrix, *T*, rely on the described torso model or the regression approaches based on measured patient data.The potential distributed on the surface of the torso obtained by performing FEM within the torso model is measured as ECG from eight selected sensors, which is later used for extracting the VCG signals using the VCG derivation approaches^36^. The VCG extraction is given by

3 $$\begin{aligned} VCG_{derived}= T. ECG_{simulated} \end{aligned}$$

First, VCG data is derived from the simulated ECG in the following way: The surface potentials generated by solving the linear equations inside the mesh are recorded on the surface using eight precordial electrodes V1 to V6, Lead I and Lead II. The standard method called Inverse Dower transform is used to extract 3-lead VCG from eight leads ECG [1]^37^.

**Fig. 3:**
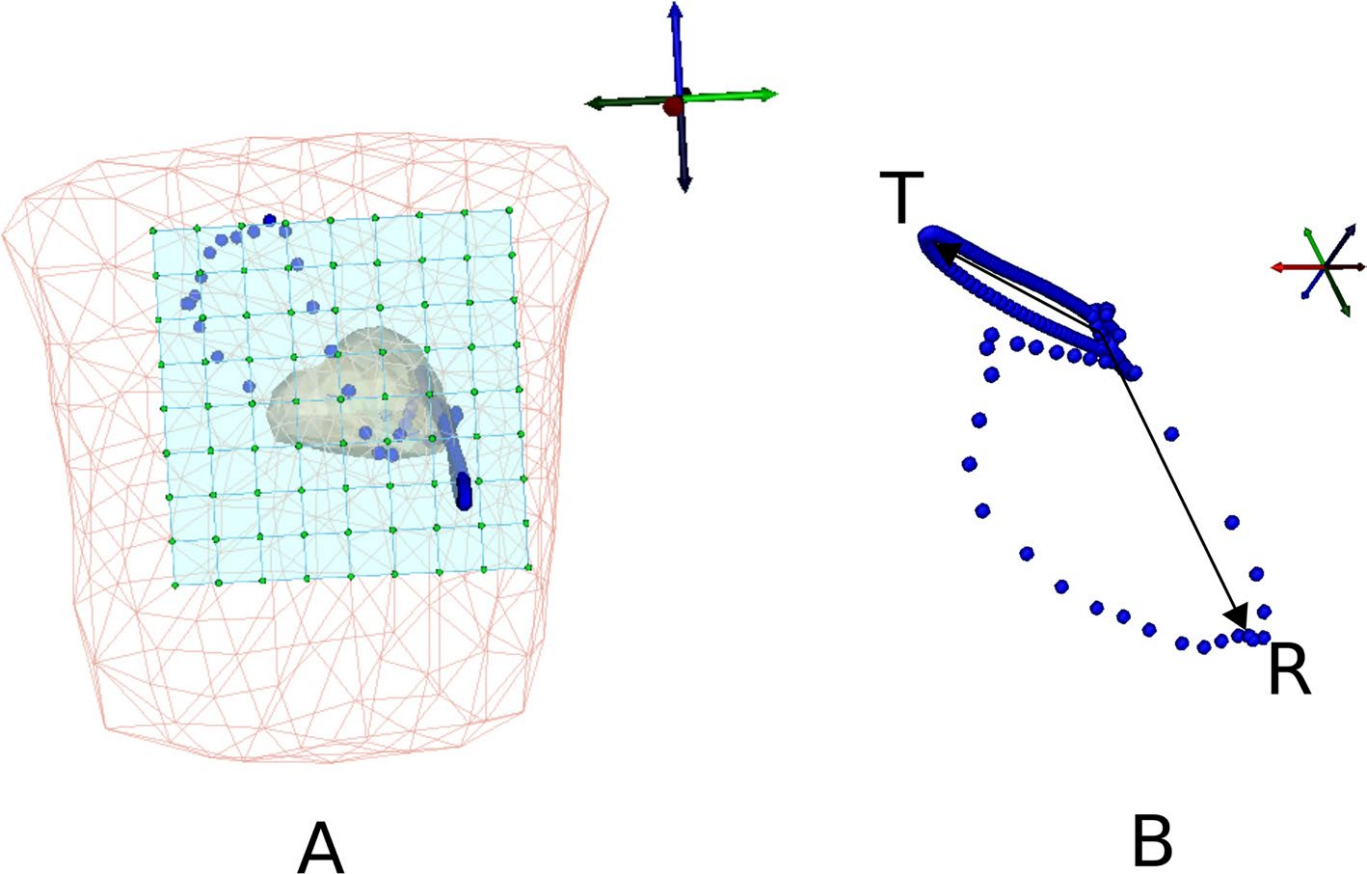
(**A**) Conceptual model of VCG (blue dots) assumed at the center of the heart surface, (**B**) Extracted spatial VCG signal from ECG.

In the literature, the heart was assumed to be an equivalent current dipole where magnitude and directions varied with time^38,39^. It is also true that each heart cell could be assumed as a tiny unit dipole with varying orientations. So, in this study, the prior assumption of dipoles at heart co-ordinates are assigned with tiny unit vectors derived from VCG. Hence, there could be a possibility to constrain the orientations of distributed dipoles from the known VCG, if the heart cells are considered. The orientations of the cardiac dipole axis are decided by the VCG loop as shown in Fig. 3B. Fig. 3A represents the assumption of VCG at the center space of heart model. The proposed transfer matrix with VCG $[v_x,v_y,v_z]$ is modeled according to Biot-savart’s law:

4 $$\begin{aligned} {\textbf{L}}_M^{uVCG} = \frac{\mu _0}{4\pi } \int \frac{{\textbf{r}}_M - {\textbf{r}}_q}{\Vert {\textbf{r}}_M - {\textbf{r}}_q\Vert ^3} \times P_0 \hat{{\textbf{e}}}^{uVCG} \, d{\textbf{v}}'. \end{aligned}$$

$P_0.\hat{{{\textbf {e}}}}^{uVCG}$ represents the unit VCG vectors assigned to the heart nodes with unit strength.

The computational steps to compute the forward problem using VCG priors are as follows:

1. Load the heart and the torso geometries spatially aligned with the MCG detectors.
2. Insert/place a dipole of strength $P_0$ at ${{\textbf {r}}}_q$ location ($q = 1$).
3. Constrain the orientation of the dipole with the unit VCG function ${\hat{v}}_q^{uVCG} = \frac{{{\textbf {v}}}_q(t)}{|{{\textbf {v}}}_q(t)|}$.
4. Rotate the dipole around its axis to complete ${{\textbf {v}}}_q=1(t)$ cycles ($t = [1, T]$) and record the corresponding field ${{\textbf {L}}}^{uVCG}$ generated at the *M* detectors (Figure 3B).
5. Shift the location of the dipole to the next adjacent heart node $\delta ({{\textbf {r}}} -{{\textbf {r}}}_q)$, constrain it with the unit VCG ${\hat{v}}_q^{uVCG}$, and record the distributed fields generated from the shifted dipole.
6. Repeat the above step at the $r_q$ positions for the time steps $t = 1$ to *T* cycles.

The SCIRun connections include loading of the spatial geometries of the heart, the torso, and the MCG sensors. In parallel to that, the dipole and the VCG signals are loaded in the network. The nodes of the discretized heart are collected using the *GetColumnOrRowFromMatrix* module and are formed as an array of vectors on which the dipole is to be set. Simultaneously, the values of the orthogonal 3D VCG signals are collected and the desired time instant is selected for the evaluation of the unit vectors. The module *CalculateFieldData* is connected to represent the input values in the form of scalars and/or vectors (in this case). After gathering the information of the electric field, the skin conductivity layers, the orientations of the dipole at the desired node & the unit VCG, the MCG sensors, the modules are connected to *SimulateForwardMagneticField*^40,41^. This module generates the magnetic field intensity at the MCG detectors due to the dipole at the heart node with the constrained orientations from the VCG. The module *GetRoworColumnMatrices* selects the step of nodes to shift the dipole positions that are assigned to on the heart. The resulting fields due to the dipoles are recorded with the help of the *WriteMatrix* module.

### Computation of MCG

**Fig. 4:**
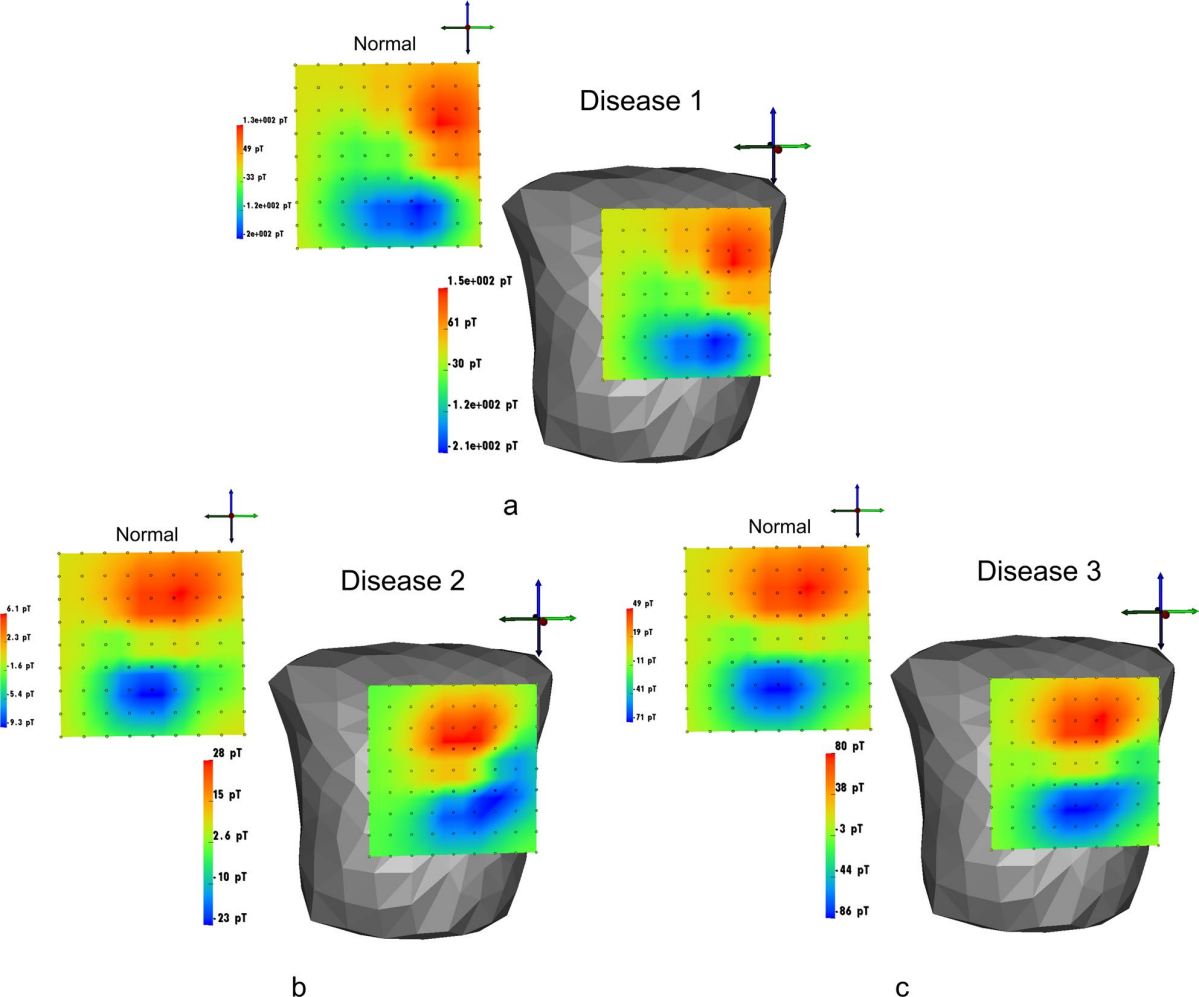
Generated magnetic field maps in SCIRUN for normal and abnormal cases: (**a**) increased R peak instant, (**b**) elevated ST instant and (**c**) increased T peak instant.

After the construction of the matrix ${\textbf{L}}^{uVCG}$, magnetic field MCG data are generated from the known epicardial potentials $s_q(t)$ at the *q* sites using the following formula:

5 $$\begin{aligned} {\textbf{B}}_{MCG}({\textbf{r}}_M, t) = {\textbf{L}}^{uVCG}({\textbf{r}}_M, {\textbf{r}}_q) \cdot {\textbf{s}}_q({\textbf{r}}_q, t) \end{aligned}$$

where $s_q(t)$ is the epicardial potentials at the $q = [1, Q]$ nodes occurring in the time duration $t = [1, T]$. These magnetic fields are considered the observed signals in the inverse problem. The ECGSIM^42,32^, developed at Radboud University Medical Center, Nijmegen, the Netherlands by A. Van Oosterom and team, is an effective tool that provides the researchers to simulate and study about the normal and the abnormal cardiac activities at the heart and the body surface potentials; allows the users to manipulate the amplitudes and duration of the heart membrane potentials. Such a model representing the heart potential distributions provides a significant prior information of the multiple source activities with respect to time. In this research work, the ECGSIM has been utilized to model and study the MI cases by rupturing a certain node on the myocardial surface. The epicardial potentials over the heart are con- sidered the distributed sources in the forward study. The MCG maps that are considered for different cases are shown in Fig. 4.

### Inverse problem: Bayesian approach

In the Bayesian estimation, the magnitudes of the unknown source activities are estimated, which are priorly assumed to be random with some known prior distributions. The joint probability density function (pdf) of magnetic field data *B* and source activities *s*, *p*(*B*, *s*) are assumed to be random. In this, *p*(*s*) is the prior pdf of *s* and $p(B\mid s)$ is the likelihood function of MCG measurement conditioned on the sources *s*. The posterior distribution to estimate the activities can be written as:

6 $$\begin{aligned} p(s\mid B)=\frac{p(B\mid s).p(s)}{p(B)} \end{aligned}$$

where *p*(*B*) in the denominator is the normalizing factor. The prior and likelihood functions are assumed to be normally distirbuted with zero mean and $\sigma ^2$ variance. The detailed model is explained in the next sections.

The epicardial potentials are denoted as $s_q(t)$ with $q\times t$ dimensions, that are assigned *q* to nodes on the surface of the heart at time *t*, $t=1,2,.. T$. The MCG data at *M* sensors is denoted as ${{\textbf {B}}}_M(t)$. In order to map the matrix from source locations to sensor locations, a geometric profile matrix called lead field *L* with dimensions $M\times q$ is introduced. It is formulated as a linear model:

7 $$\begin{aligned} {\textbf{B}}_M(t)={\textbf{L}}_M^q \cdot {\textbf{s}}_q(t)+n(t) \end{aligned}$$

where *n*(*t*) is the noise in the MCG data.

#### Prior selection: Gaussian distribution

The prior distribution of sources in the exponential form can be written as:

8 $$\begin{aligned} p(s)=\frac{1}{N_s(\alpha )}\exp (-\alpha E_s) \end{aligned}$$

and the denominator $N_s(\alpha )$ is the normalizing constant with $\alpha$ assumed as hyper-prior and is defined as:

$N_s(\alpha ) =(\frac{2\pi }{\alpha })^{\frac{Q}{2}}$ which ensures that $\int p(s)ds$ has unit area, The weight prior $E_s =\frac{1}{2}\sum _{j=1}^{Q}s_j^2$ is a function that controls the type of prior distribution assumed. In the work, the distribution is assumed to be Gaussian with zero mean and variance $\sigma ^2=\frac{1}{\alpha }$ and can be written as:

9 $$\begin{aligned} p(s)=\frac{1}{(\frac{2\pi }{\alpha })^{\frac{Q}{2}}}\exp \left( -\frac{\alpha }{2}\vert s \vert ^2\right) \end{aligned}$$

#### Likelihood function

The probability density function (pdf)of MCG given the sources can be expressed as:

10 $$\begin{aligned} p(B\mid s)=\frac{1}{N_B(\beta )}\exp (-\beta E_B) \end{aligned}$$

$N_B(\beta )$ is the normalizing constant with $\beta$ as hyper-prior parameter assumed and defined as:$N_B(\beta ) =(\frac{2\pi }{\beta })^{\frac{M}{2}}$ The distribution of the measured data is assumed to be Gaussian with zero mean and $\sigma ^2=\frac{1}{\beta }$

The expectation of MCG is calculated using sum of squares error function which leads to the following likelihood function:

11 $$\begin{aligned} p(B\mid s)= \frac{1}{(\frac{2\pi }{\beta })^\frac{M}{2}}\exp \left( -\frac{\beta }{2}\vert |B-Ls\vert |^2\right) \end{aligned}$$

#### Posterior probability

By utilizing the likelihood and prior distributions, Bayes’ theorem is used to construct the posterior.

12 $$\begin{aligned} p(s\mid B)=\frac{1}{N_K(\alpha ,\beta )}\exp \left( -K(s,\alpha ,\beta )\right) \end{aligned}$$

$N_K(\alpha ,\beta )$ is the normalizing factor and the above equation can be expanded as:

13 $$\begin{aligned} p(s\mid B)=\frac{1}{N_K(\alpha ,\beta )}\exp \left( -(\epsilon _s+E_B)\right) \end{aligned}$$

By equating the powers of the prior, likelihood and the posterior,

14 $$\begin{aligned} -K(s,\alpha ,\beta )=-\beta E_B-\alpha \epsilon _s \end{aligned}$$

By differentiating wrt s, we get

15 $$\begin{aligned} {{\textbf {s}}}_{MP}=\left( L^TL+\frac{\alpha }{\beta }I \right) ^{-1}{{\textbf {L}}}^T.{{\textbf {B}}} \end{aligned}$$

$s_{MP}$ is the MAP estimate of the prior sources *s*.

#### Defining hyper-parameters $\alpha$ and $\beta$

The posterior distribution of the source amplitudes is given by

16 $$\begin{aligned} p(s\mid B)=\int \int p(s,\alpha , \beta \mid {{\textbf {B}}}) d\alpha d\beta \end{aligned}$$

The expansion of the above equation gives:

$$\begin{aligned} =\int \int p(s\mid \alpha \beta ,{{\textbf {B}}}) p(\alpha ,\beta \mid {{\textbf {B}}}) d\alpha d\beta \end{aligned}$$

The posterior distribution of $p(\alpha ,\beta \mid {{\textbf {B}}})$ is assumed to be sharply peaked at maximum probable values of $\alpha _{MP}$ and $\beta _{MP}$. So,

17 $$\begin{aligned} p(s\mid B) = p(s\mid \alpha _{MP}, \beta _{MP},{{\textbf {B}}}) \end{aligned}$$

Now, the posterior of inner integral is evaluated to find the hyperparameters $\alpha$ and $\beta$:

18 $$\begin{aligned} p(\alpha _{MP},\beta _{MP})=\frac{p({{\textbf {B}}}\mid \alpha ,\beta )p(\alpha ,\beta )}{p({{\textbf {B}}})} \end{aligned}$$

$p(\alpha ,\beta )$ is called as hyper-prior distribution. The above equations represents the Bayesian analysis in a hierarchical manner i.e., first estimating the distribution of s and then finding the optimum value of hyper-parameters These hyper-priors are non-informative and assumed to be flat. Since the denominator is independent of $\alpha$ and $\beta$, it is more convenient to maximize $p(B\mid \alpha ,\beta )$ which leads to maximizing the posterior $p(\alpha ,\beta \mid B)$. By performing the evidence approximation method to find out $\alpha$ and $\beta$ as explained in^19,20^, the solution reaches to the following equations:

19 $$\begin{aligned} & \alpha = \frac{\gamma }{2E(s_{MP})} \end{aligned}$$

20 $$\begin{aligned} & \beta = \frac{M-\gamma }{2E_B} \end{aligned}$$

where $\gamma =\sum _{i=1}^{J}\frac{\lambda _i}{\lambda _i+\alpha }$ with $\lambda _i$ being the eigen values of $\beta L^TL$ An iterative algorithm (Algorithm 1) is presented with the help of above equations to get optimum $\alpha$ and $\beta$ values that estimates source amplitude $s_{MP}$.

**Algorithm 1:**
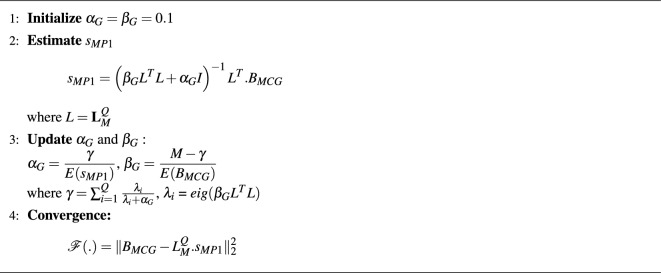
Bayesian Approach using Gaussian Distribution.

The above algorithm works assuming the priors to be normally distributed. So, the distribution functions have smooth shaped curves at their peaks. Due to this character, the above algorithm yields Tikhonov type regularization ($L_2$ norm) in the deterministic approach.

Sometimes, smoother solutions formed using Gaussian priors when mapped on to heart’s surface show unnecessary localization scattering, which may frame the normal cell activities to be abnormal. In order to avoid such situations, the prior that produce sparse solutions can be employed. The prior that is capable of producing such sparse solution is Laplacian distribution. This prior forces the unnecessary source estimates to zero making the solutions sparse.

#### Prior selection: Total variation

The formulation of $L_1$ norm regularization is equivalent to usage of a Laplacian prior model in Bayesian approach. The corresponding weight prior $\epsilon _s$ and its distribution *p*(*s*) can be modeled as:

21 $$\begin{aligned} & \epsilon _s=\sum _{j=1}^{Q}\vert s_j \vert \end{aligned}$$

22 $$\begin{aligned} & p(s)=\frac{1}{\left( \frac{2}{\alpha '}\right) ^Q}\exp (-\alpha '\vert s \vert ) \end{aligned}$$

where $\alpha '$ is the hyperparameter The MAP estimate of this problem reaches the cost function:

23 $$\begin{aligned} \min _s\vert |B-Ls|\vert +\lambda \vert s\vert _1 \end{aligned}$$

**Fig. 5:**
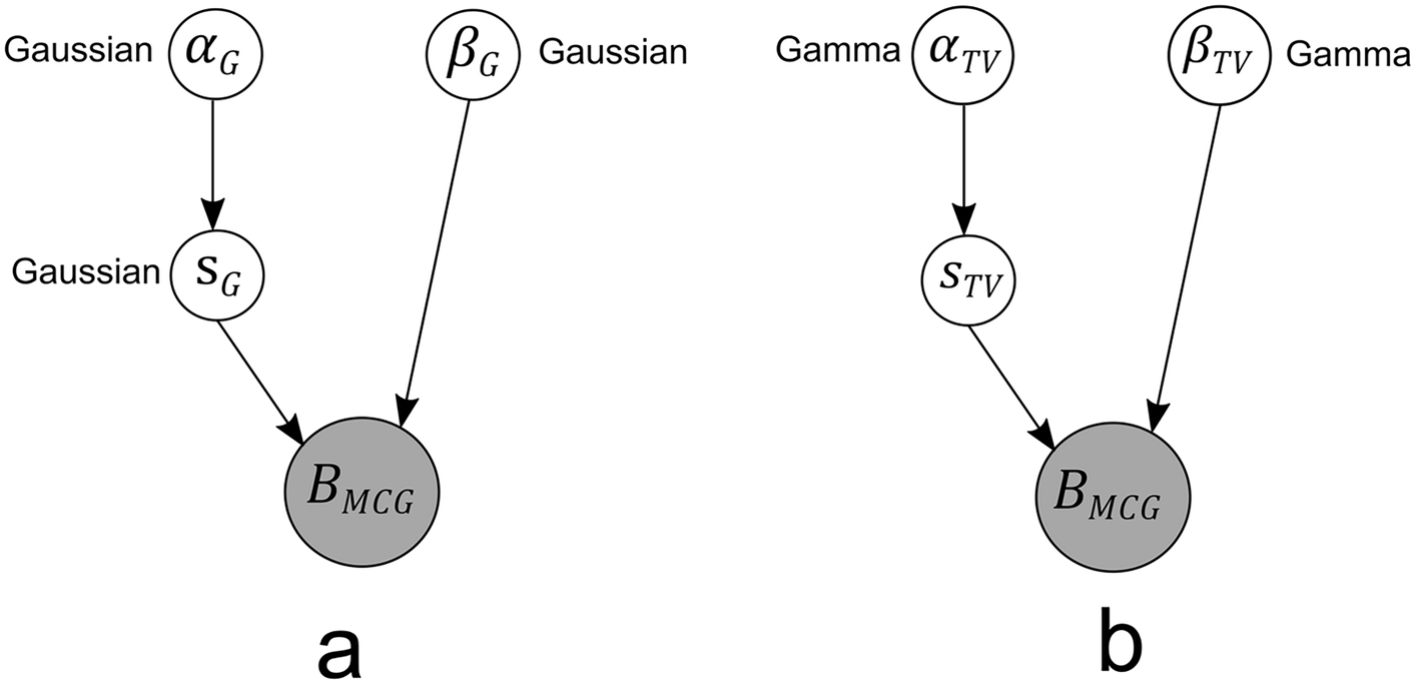
Grahical models of Bayesian frameworks with (**a**) Gaussian prior, (**b**) TV prior.(Gray: Known variables, White: Unknown variables).

Solving the above prior distribution is complicated and since it reaches $L_1$ norm, has no closed form (not differentiable at the origin). The other reason is that the prior is not conjugate to the likelihood condition of Normal distribution and Laplacian prior would not lead to tractable Bayesian solution^25^. In order to solve this type, hierarchical priors can be used^26^. Figure 5a and b represent the graphical acyclic graphs of joint probability Bayesian models from Gaussian and TV priors respectively..

### Hierarchical Bayesian inference using total variational prior

As defined earlier, the same transfer matrix $(M\times Q)$ with *M* sensors and *Q* sources are considered in solving the inverse method using TV priors. TV regularization is a non-quadratic method which uses $L_1$ norm penalty of the gradient function and produces sparse results. In this work, normal gradient of the heart potentials are considered that bases the $L_1$ norm regularizer.

The likelihood distribution of the MCG data is same as the previous method since it is assumed to obey Gaussian distribution (see equation 17). The TV prior model for *Q* sources is given by:

24 $$\begin{aligned} p(s\mid \alpha )\propto \alpha ^{\frac{Q}{2}}\exp (-\alpha TV(s)) \end{aligned}$$

where $$TV(s)=\sum _{i}^{Q}\sqrt{(D_{s_i})^2}$$

where $D_{s_i}= \frac{\partial s }{\partial n}$ is the spatial derivatives of the heart surface, $\alpha$ is the hyperparameter assumed to be known that estimates the source distributions.

The joint posterior distribution with hyperparameters $\alpha$ and $\beta$ can be written as:

25 $$\begin{aligned} p(\alpha , \beta ,s, B)= p(\alpha ) p(\beta ) p(s\mid \alpha ) p(B\mid s,\beta ) \end{aligned}$$

where the hyper-prior $\alpha$ (inverse variance of sources) helps to determine $p(s\mid \alpha )$ and $\beta$ (inverse variance of field data) helps in determining $p(B\mid s, \beta )$.

#### Hyperparameters

Designing the hyperparameters is little complicated than the previous method. The hyperpriors are modeled as Gamma distributions. The reason is that this distribution makes the conjugate prior for the inverse variance of the Gaussian priors. The gamma priors of $\alpha$ $\beta$ are defined as:

26 $$\begin{aligned} & \Gamma (\alpha \mid a_{\alpha _0},b_{\alpha _0}) \propto \alpha ^{a_{\alpha }^0-1} \exp (-\alpha b_{\alpha }^0) \end{aligned}$$

27 $$\begin{aligned} & \Gamma (\beta \mid a_{\beta _0},b_{\beta _0}) \propto \beta ^{a_{\beta }^0-1} \exp (-\beta b_{\beta }^0) \end{aligned}$$

In order to approximate the inference, posterior distribution is evaluated. But, the joint pdf $p(\alpha , \beta ,s\mid B)$ cannot be found in a closed form. As suggested in^27^, variational methods are use to approximate the posterior pdf to the new assumed pdf $q(\alpha ,\beta ,s)$. This can be done using Kullback-Leibler (KL) divergence minimization method where variational criteria is used to find $q(\alpha , \beta ,s)$. KL divergence is defined as:

$$\begin{aligned} KL(q(\alpha ,\beta ,s)\vert |p(\alpha ,\beta ,s\mid B))= \end{aligned}$$

28 $$\begin{aligned} \int _{\alpha ,\beta ,s}q(\alpha , \beta ,s).\log \left( \frac{q(\alpha ,\beta ,s) }{p(\alpha ,\beta ,s\mid B)}\right) d\alpha d\beta ds \end{aligned}$$

The meaning of the above equation is to approximate the distribution *q*() from a distribution *p*() and hence minimizing the similarity between the distributions. The final expression becomes:

29 $$\begin{aligned} KL(q\vert |p)=-{\mathscr {L}}+\log p(B) \end{aligned}$$

The difference between *log* probability of MCG data and the KL divergence forms the lower bound ${\mathscr {L}}$. This means, the lower bound meets the *log* probability of observation data *iff* the estimate becomes equal to the true posterior distributions. Now, to define the posterior model, following method is used. As mentioned in^20^, inequality is assumed for stating the two variables, $g\ge 0$ and $h\ge 0$, such that

$$\begin{aligned} \sqrt{g} \le \frac{g+h}{2} \end{aligned}$$

A *Q* dimensional vector $v_i\in \Re ^Q$ for $i=1,2... Q$ is assumed such that the model can be expressed as:

30 $$\begin{aligned} {\mathcal {M}}(\alpha ,s,v) =\alpha ^\frac{Q}{2} \exp \left( -\frac{\alpha }{2} \sum _{i} \frac{(D_{s_i})^2+v_i}{\sqrt{v_i}} \right) \end{aligned}$$

The above model is defined from $g=(D_{s_i})^2$ and $h=v_i$ If the equation is compared with the prior model and by utilizing the inequality:

$$\begin{aligned} p(s\mid \alpha )\propto c.\alpha ^{\frac{Q}{2}}\exp (-\alpha \sum _{i} (D_{s_i})^2) \end{aligned}$$

it can be inferred that:

31 $$\begin{aligned} p(s\mid \alpha )\ge c.{\mathcal {M}}(\alpha ,s,v) \end{aligned}$$

and

$$\begin{aligned} & p(s\mid \alpha )\ge p(\alpha )p(\beta ){\mathcal {M}}(\alpha ,s,v). p(B\mid s,\beta ) \\ & \equiv G(\alpha ,\beta ,s,v,B)\\ & KL(q\vert |p)=\min \int _{\alpha ,\beta ,s}q(\alpha , \beta ,s).\log \left( \frac{q(\alpha ,\beta ,s)}{G(\alpha ,\beta ,s,B)}\right) \\ & +\log p(B) \end{aligned}$$

The above equations written in the simple form are

32 $$\begin{aligned} q^c(s)=\min _{q(s)}\left[ KL(q\vert |p)\right] \end{aligned}$$

and

33 $$\begin{aligned} v^{c+1}=\min _v\left[ KL(q\vert |p)\right] \end{aligned}$$

By differentiating the above equation wrt *q*(*s*) and *v* for iterations count *c* and set the gradient to zero,

for $$\hat{s_Q}:$$

34 $$\begin{aligned} E(q^c(s))=\hat{s_Q}^c=cov(\hat{s_Q}^c).{\hat{\beta }}L^T B \end{aligned}$$

where $$cov({\hat{s}}_Q^c)=\left( L^T{\hat{\beta }}L+{\hat{\alpha }}D^TW(v^c)D\right) ^{-1}$$

for $$v^c:$$

35 $$\begin{aligned} v^{c+1}=\sum _{i=1}^{Q}(D_{s_i})^2 \end{aligned}$$

**Algorithm 2:**
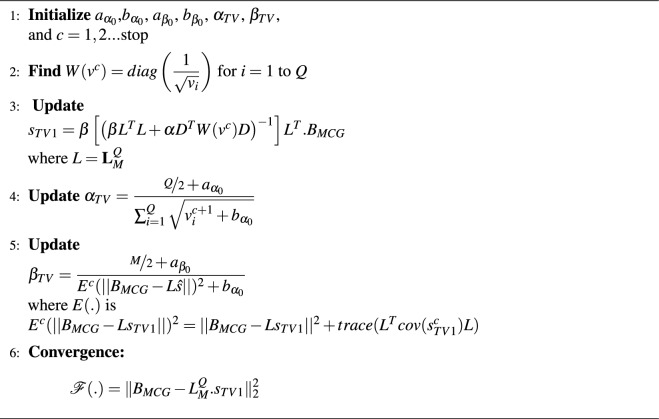
Hierarchical Bayesian Inference with Total Variation Prior.

### Bayesian solution: VCG based Gaussian prior

Gaussian priors are used for VCG estimation. In both the conventional algorithms 1 and 2, the lead field used were static, where the orientations of the prior dipoles were tangential to the detector planes. In this paper, variational lead fields are proposed and constructed based on unit VCG signals and is used in the inverse algorithms using Bayesian approach called as VCG based Gaussian and TV priors.

The bayesian model with orientation is defined as:

36 $$\begin{aligned} p(e|L)=\frac{p(L|e). p(e)}{p(L)} \end{aligned}$$

where posterior *p*(*e*|*L*) relies on the variational transfer matrix containing distributed VCG vectors as priors *p*(*e*). The distribution corresponding to the prior orientation model is given by:

37 $$\begin{aligned} p\left( e_{q}^{u V C G}\right) \propto \frac{1}{\left( 2 \pi / \alpha _{VCG}\right) ^{{Q}/{2}}} \cdot \exp \left( \frac{-\alpha _{VCG}\Vert e\Vert ^{2}}{2}\right) \end{aligned}$$

**Algorithm 3:**
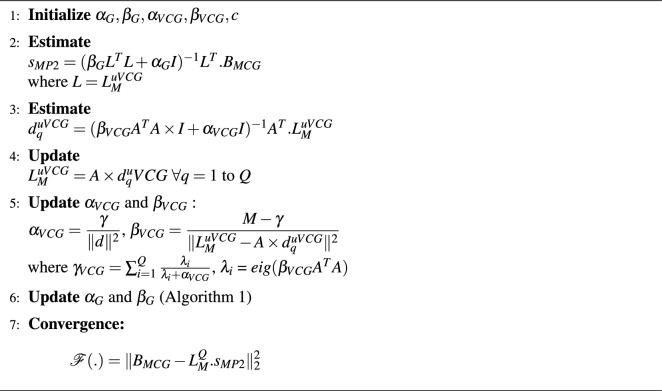
Bayesian Inference Framework Using VCG-based Gaussian Prior.

**Fig. 6:**
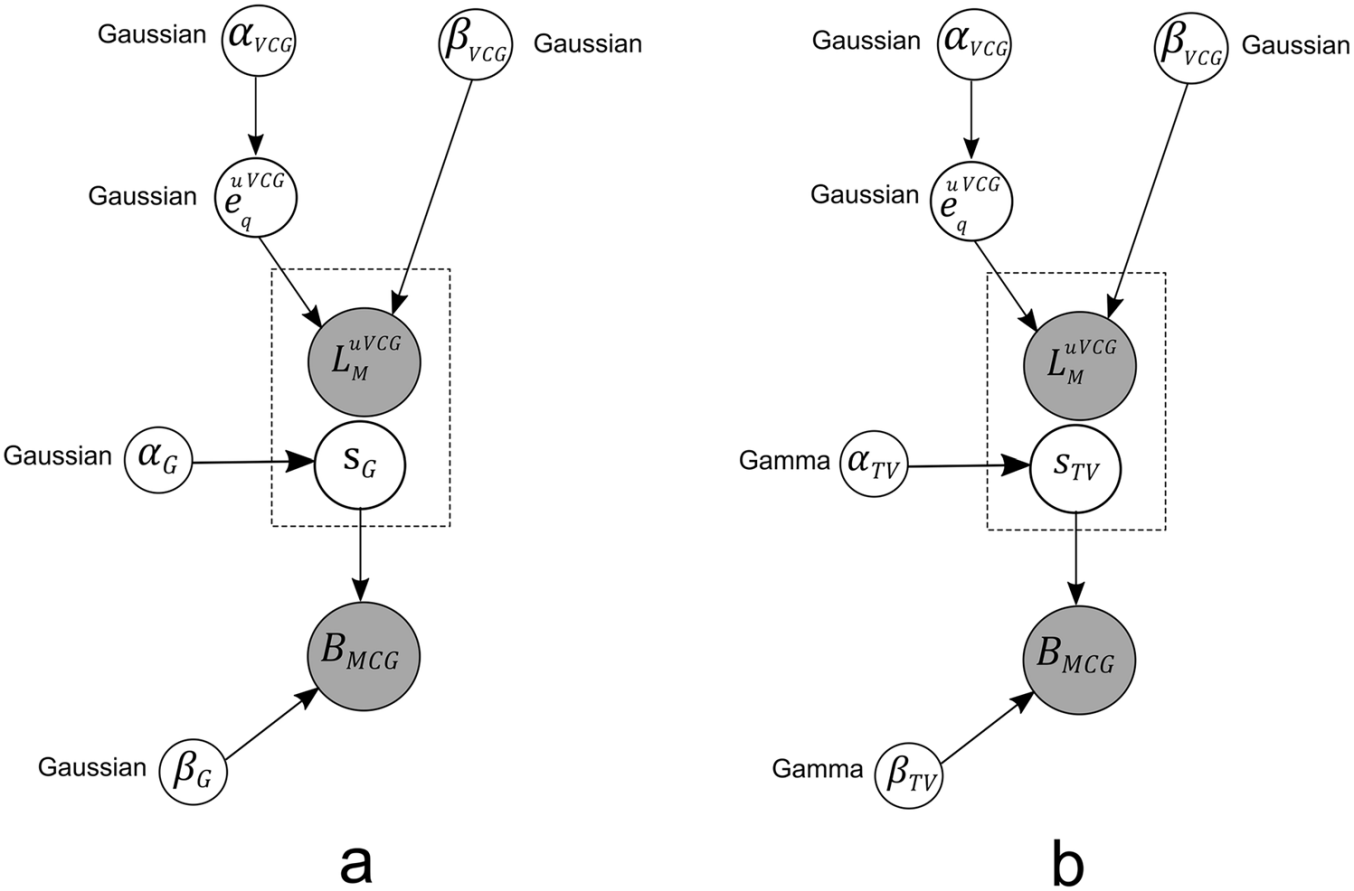
Graphical models of Bayesian frameworks with (**a**) VCG based Gaussian, and (**b**) VCG based TV prior. (Gray: Known variables, White: Unknown variables).

Insertion of VCG based orientation priors are compiled with TV strength priors. The unit VCG signals are assumed to be normally distributed in both Algorithms 3 and 4.The above algorithm explains the bayesian estimation of cardiac sources by considering the tiny orientations (uVCG) into account. This updates the transfer matrix in each iterations which contains constrained estimated orientations derived from unit VCG on the desired source locations. The graphical models of proposed algorithms are illustrated in Fig. 6a and b which used VCG priors with variational transfer matrices $L_M^{uVCG}$ in the Bayesian approximation.

###  Bayesian solution: VCG based TV prior

In the previous algorithm, the VCG priors were assumed to be Gaussian pdf while updating the dynamic transfer matrices. The estimation of the VCG vectors with latencies generated smoother results due to the Normal pdfs of the hyper-priors $\alpha$ and $\beta$. To improve the sparsity nature, the hyper-priors for sources and MCG observations are designed to be fit with Gamma distributions while VCG priors are assumed to Gaussian distributed. Algorithm 4 explains the implementation of Bayesian inverse solutions estimated with the use of VCG and hyper-priors to update the dynamic transfer matrix in each iteration; which converges to $s_{TV2}$ where the cost function between observed and simulated measurements reaches minimum.

**Algorithm 4:**
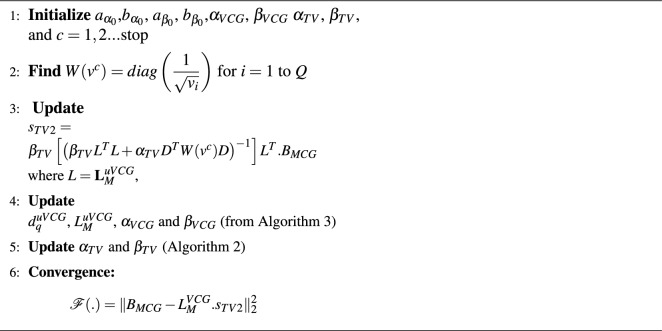
Bayesian Inference Framework Using VCG-based TV Prior.

**Fig. 7:**
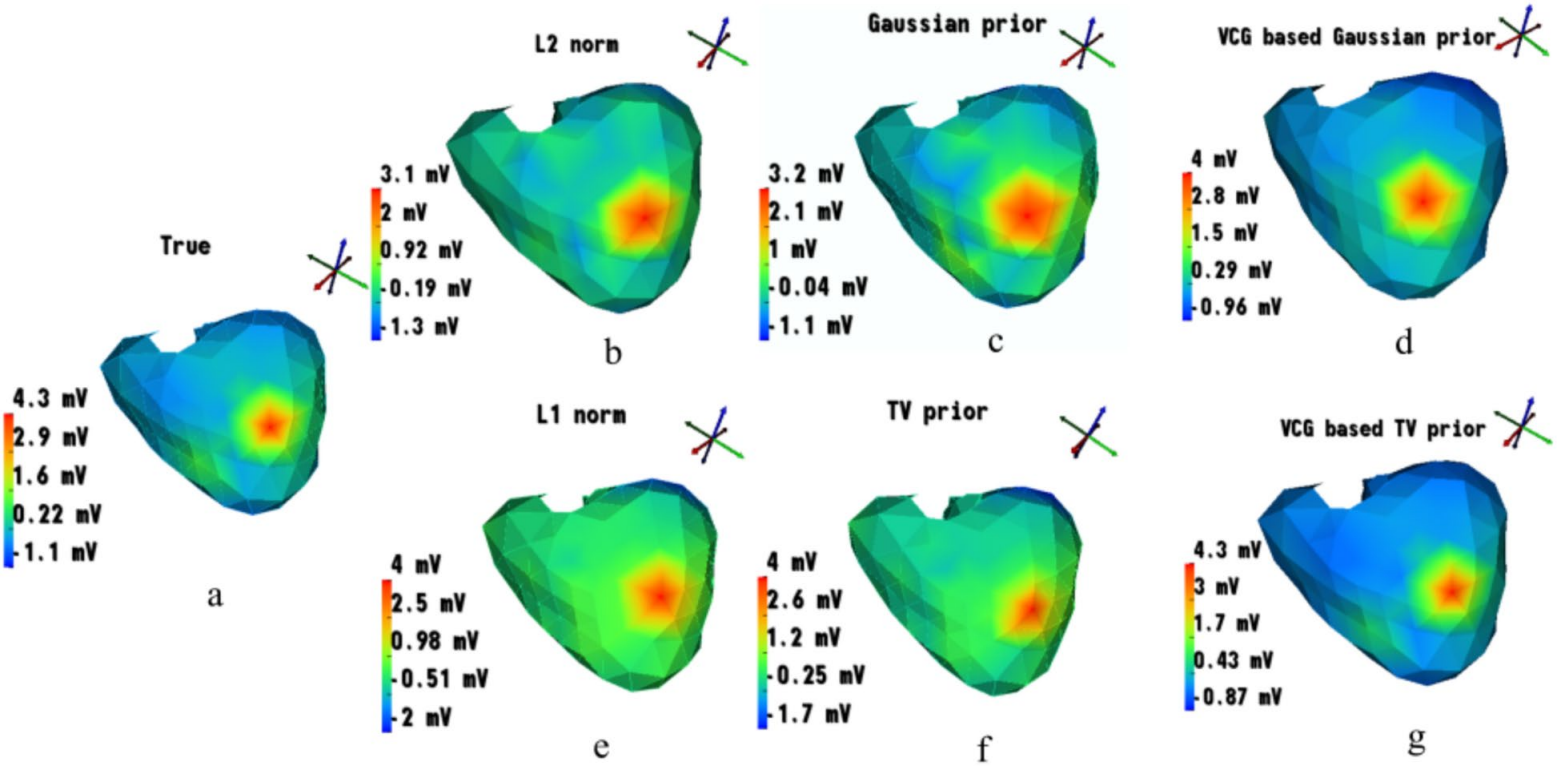
Comparison of visualization maps of different algorithms with true epicardial potentials at $t = 18ms$. (**a**) True source potentials, (**b**) Standard Tikhonov ($L_2$ norm) regularization, (**c**) Bayesian regularization with Gaussian prior (Algorithm 1), (**d**) Bayesian regularization with Gaussian and VCG priors (Algorithm 3), (**e**) $L_1$ norm regularization, (**f**) Bayesian regularization with TV prior (Algorithm 2), (**g**) Bayesian regularization with TV and VCG priors (Algorithm 4).

## Results and discussion

The results are compared with two factors: i. The mean squared error between the true Fig. 7a and the estimated epicardials and, ii. Based on the region spread. Fig. 7b is the reconstructed epicardial potentials from the standard Tikhonov regularization technique. It was observed from the spread range at the *t* instant varied with a markable difference from that of the true epicardial map. This method usually produces smoothness in the maps, thus could not border out the desired abnormal region. From Fig. 7e, the results are obtained using the $L_1$ norm method which has a more sharper spread than the latter methods, depicting a sparse nature. Since both the $L_1$ and $L_2$ regularizers are deterministic, they somehow lack their ability in reconstructing the sources during uncertain conditions. Bayesian statistics promise to overcome the situation in cases of uncertainty and inconsistency of the epicardial sources and the MCG channels.

Since the algorithm uses a Gaussian prior of sources, the range in the estimated maps is improved than that of the $L_2$ and $L_1$ norms; but one can observe a spread in the desired region (producing smoother results) as in Fig. 7c. The sparse nature of the results can be obtained with the help of Algorithm 3, where the TV priors play a significant role (shown in Fig. 7f). Figure 7g reconstructs the epicardial sources with a VCG prior-based Bayesian result where, visually, the ranges in the maps are almost identical to that of the true one. This is due to the estimation of the VCG points at each and every iteration that drives to reach an optimum region of spread effectively. It is noted that the results of uncertainties are addressed by the Bayesian results (Fig. 7d and g) with an applied SNR of 50 dB to the measured/observed signals.

The performance of the proposed algorithms for MCG source localization is compared with the standard Tikhonov regularization with noise-free and noise conditions in terms of MSE. The average MSE obtained for the Tikhonov approach in the noise-free condition was 0.43 mV. The other deterministic method, the $L_1$ norm, provided a slight improvement of 0.22 mV in MSE than the $L_2$ regularization. When compared to the Bayesian methods, the MSE was 0.37 mV and 0.18 mV for the Gaussian priors (Algorithm 1) and the TV priors respectively; which showed an improvement of 16–22% in performance compared to the deterministic methods in the noise-free conditions. The proposed algorithms (Algorithms 3 and 4) estimated both the VCG orientations and the epicardial potentials due to the dynamic lead fields.

**Fig. 8:**
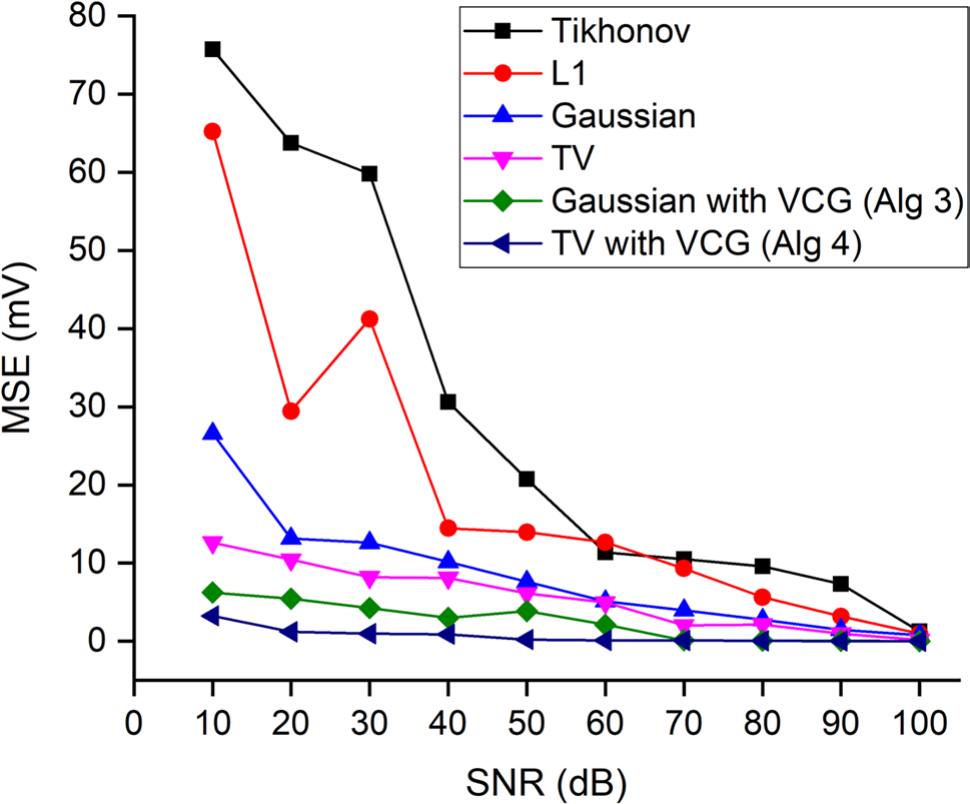
Comparison of MSE measures between true and estimated epicardials.

The average MSE dropped to 0.13 mV and 0.0873 mV for the Bayesian approximations with the VCG-based Gaussian priors and the VCG-based TV priors respectively. The performances of the algorithms when subjected to noise are illustrated in Fig. 8. The deterministic approaches with 10 dB SNR yielded an average MSE of 60–75 mV. The MSE values dropped drastically to the 20–30 mV range for the Bayesian methods (Algorithm 1) which solved the problem of the uncertainty nature at 10 dB SNR. The MSE reached approximately 12 mV at 10 dB SNR for the TV prior approach due to the sparse nature that cancels out the smoothness produced in the previous method. It can be observed from Fig. 8 that Algorithms 3 and 4 obtained the least MSE values ranging from 0.03–5 mV at SNRs 10–100 dB.

The usage of the VCG vectors and the dynamic transfer matrices significantly boost the system’s performance. This is because the approximation of the VCG priors affects directly the estimation of the magnetic field maps at the observation plane. Since the static lead field was used in the conventional inverse methods, the solution may cause an error in the measurements if there persists any deviation of the pointed cardiac vectors assumed in the construction of the transfer matrix. The proposed approach hence used the VCG priors along with the epicardial priors that promises a good solution to the ill-posed problem as the orientations are considered as a parameter while preparing the Bayesian models.

To evaluate the orientation performances in Algorithm 3 and Algorithm 4, the angle of deviations ($\theta$) are measured between the true dipole ($\hat{{{\textbf {e}}}}_q^{uVCG}$) and the estimated vector ($\hat{{{\textbf {d}}}}_q^{uVCG}$):

38 $$\begin{aligned} \theta =\cos ^{-1}\left( \frac{\hat{{{\textbf {e}}}}_q^{uVCG}. \hat{{{\textbf {d}}}}_q^{uVCG}}{\Vert \hat{{{\textbf {e}}}}_q^{uVCG}\Vert \Vert \hat{{{\textbf {d}}}}_q^{uVCG}\Vert }\right) \end{aligned}$$

**Table 1:** Angle of deviation for VCG based Gaussian (Alg. 3) and TV priors (Alg. 4).

Condition	Vectors	$\theta _G$			$\theta _{TV}$		
		D1	D2	D3	D1	D2	D3
Noise-free	$\hat{{{\textbf {e}}}}_q^{uQ}$	2.33	1.97	2.14	0.62	0.31	0.88
	$\hat{{{\textbf {e}}}}_q^{uR}$	1.56	2.45	1.62	0.47	0.61	0.15
	$\hat{{{\textbf {e}}}}_q^{uS}$	1.24	3.21	2.77	0.28	0.3	0.92
	$\hat{{{\textbf {e}}}}_q^{uT}$	0.67	1.58	0.56	0.63	0.55	0.14
Noise: 10dB	$\hat{{{\textbf {e}}}}_q^{uQ}$	8.26	7.56	6.29	1.85	2.06	1.84
	$\hat{{{\textbf {e}}}}_q^{uR}$	3.56	4.62	9.33	5.24	3.96	1.28
	$\hat{{{\textbf {e}}}}_q^{uS}$	7.18	6.32	7.64	3.66	2.58	2.55
	$\hat{{{\textbf {e}}}}_q^{uT}$	5.56	7.48	5.58	2.58	5.14	5.64
Noise: 20dB	$\hat{{{\textbf {e}}}}_q^{uQ}$	6.25	5.82	4.25	3.65	1.85	2.79
	$\hat{{{\textbf {e}}}}_q^{uR}$	3.65	4.65	5.66	2.36	1.91	1.52
	$\hat{{{\textbf {e}}}}_q^{uS}$	8.52	7.63	8.21	5.36	2.51	2.68
	$\hat{{{\textbf {e}}}}_q^{uT}$	7.64	8.32	3.68	3.21	3.54	1.31
Noise: 50dB	$\hat{{{\textbf {e}}}}_q^{uQ}$	5.32	6.38	5.19	2.35	1.67	2.56
	$\hat{{{\textbf {e}}}}_q^{uR}$	2.56	4.22	6.46	3.14	2.66	2.38
	$\hat{{{\textbf {e}}}}_q^{uS}$	6.32	7.31	8.54	4.36	3.2	1.17
	$\hat{{{\textbf {e}}}}_q^{uT}$	4.76	3.94	3.60	3.78	4.31	2.65

Table 1 represents the results of deviation between the true and the estimated VCG vectors from Algorithm 3 and 4. The minimum angle of deviation for disease D2 was 1.97° in noise-free condition and 3.94° for 50 dB noise in the measurements both at *S* instant (node index 3 on the heart surface). The TV prior with VCG algorithm showed and improvement of 2.91° and 4.11° for D2 case at *S* instant respectively. In order to understand the region of infarctions on map, the reconstructed borders of inverse solutions are evaluated in terms of region of spread (ROS). It is defined as the spatial distance between the centre point source (${{\textbf {R}}}_c: x_c,y_c,z_c$) and the surrounding neighbouring sources (${{\textbf {R}}}_i: x_i,y_i,z_i \, \forall i=1$ to *n* ) where *n* is the collection of abnormal points (selected based on the border spread surrounding of abnormal targeted node). The equation used to compute spread is:

39 $$\begin{aligned} ROS=\sum _{i=1}^{n} \frac{\left( {{\textbf {R}}}_c-{{\textbf {R}}}_i\right) ^2}{n} \end{aligned}$$

Table 2 depicts the ROS comparison formed in different cases with noise and noise-free conditions. It can be noted that for 50 dB SNR, the deterministic approach produced an average spread of 5.89-7.11 cm surrounding the abnormal node for deterministic approaches. The spread decreased to an average of 2.94-3.69 cm when it comes to Bayesian methods (Algorithm 1 and 2) at 50 dB SNR. It was observed that usage of VCG in the inverse problem provided a decrease in the average ROS error to 0.25 cm from true radius.The average spread resulted for deterministic methods were around 3.54 cm for D1, 4.36 cm for D2, and 4.29 cm for D3 at 10 dB SNR. Bayesian inference algorithm with VCG priors showed a decreased region spread to 3.03 cm for D1, 3.65 cm for D2, and 3.82 cm for D3 at 10dB SNR. In VCG based Total variation algorithm, the lowest spread was observed with 2.93 cm for D1, 3.47 cm for D2, and 3.75 cm for D3 at 10dB SNR.

**Table 2:** Region of spread (ROS) comparison in noise and noise-free conditions. (Radius of true abnormality spread: 2.5 cm).

Condition	Cases	Deterministic		Bayesian		Bayesian with VCG	
		$L_2$	$L_1$	Alg. 1	Alg. 2	Alg. 3	Alg. 4
Noise-free	D1	3.27	2.61	2.87	2.65	2.53	2.41
	D2	4.63	3.95	3.23	2.84	2.59	2.33
	D3	3.96	3.54	3.16	2.77	2.62	2.46
Noise SNR:10 dB	D1	3.54	3.45	3.26	3.12	3.03	2.93
	D2	4.36	4.25	4.13	3.88	3.65	3.47
	D3	4.29	4.14	4.04	3.93	3.82	3.75
Noise SNR:20 dB	D1	4.46	4.35	4.24	4.09	3.95	3.77
	D2	4.31	4.24	4.14	4.06	3.92	3.85
	D3	4.56	4.45	4.26	4.12	3.93	3.73
Noise SNR:50 dB	D1	6.56	5.79	4.26	3.82	2.93	2.73
	D2	8.46	7.65	5.64	3.69	3.15	2.67
	D3	6.31	4.24	3.94	3.56	3.02	2.85

## Computational efficiency and feasibility

In high dimensional settings, the advanced versions of Bayesian and total variation algorithms tends to be computationally complex. To reduce the complexity, the sparsity of the prior information was The regularization parameters in algorithms 3 and 4 with Gaussian and total variation priors elevate the solutions that are sparse or smooth. This helps in dimensionality reduction by setting constraints on the solution space focusing more on probable configurations, instead of exploring all possible solutions.

The second step in Algorithm 3, $\beta _G L^TL+\alpha _G I$ acts as regularization term that avoids large variations in the solution $s_{MP2}$. This assures that most significant dipoles effect on to the inverse solution, thereby imposing sparsity. Further, step 3 updates the dipole orientations based on unit VCG ensures that the forward transfer matrix remains compact and synchronized with the sparse source configurations. In contrast to that, algorithm 4 has the term $D^T W(v_c)D$, introduces constraints for spatial smoothness that results in smooth solutions retaining the region of source activities with larger variations while reducing noise.

**Table 3:** Computational complexity for forward problems simulated in SCIRUN software.

Forward problem	Modules in SCIRUN	Execution time (s)
Conventional	34	26.47
Proposed	39	38.25

Table 3 presents the complexity and execution times for solving conventional and proposed forward problems with count of modules in SCIRUN. The comparison execution times between different inverse algorithms are represented in Table 4. The execution times are indicated in seconds. The conventional $L_2$ norm method was found to be the fastest, while the other algorithms with VCG has obtained significantly higher execution times.

**Table 4:** Execution times resulted from different inverse algorithms.

Inverse problem	Execution time (s)
Conventional – $L_2$ norm	26.47
Conventional – $L_1$ norm	38.25
Algorithm 1	29.32
Algorithm 2	32.16
Algorithm 3 (with VCG)	51.78
Algorithm 4 (with VCG)	56.35

## Conclusion

The novelty of the study is to reconstruct the cardiac source activities by considering the VCG signal while constructing the forward problem with the VCG. In this study, the cardiac sources were imaged non-invasively as a location wise dipole with the epicardial activities on the myocardium from the magnetic field data (MCG). In this paper, novel VCG based Bayesian algorithms are modeled to reconstruct the abnormal epicardial activations. Our algorithms used unit VCG orientations in the construction of transfer matrices, thereby designing the forward problem dynamic in nature.

The introduction of unit VCG priors in the statistical inverse problem not only addresses uncertainty issues but points out the orientations of reconstructed sources. The estimation of unit vectors of VCG is needed in order to understand and visualize the current flow directions that directly affects the MCG maps at the detector level. A slight deviation in the dipole orientation may lead to larger variations in the observation plane. The current study is compared with that of conventional methods which used only static transfer matrices and unit vector approach.The outcome of this research study helps doctors to keep the track of abnormal electrical pathways of the heart without performing the invasive procedures. The novelty of the study is to reconstruct the cardiac source activities by considering the VCG signal while constructing the forward problem with the VCG. In this study, the cardiac sources were imaged non-invasively as location-wise dipoles with the epicardial activities on the myocardium from the magnetic field data (MCG). In this paper, novel VCG-based Bayesian algorithms are modeled to reconstruct abnormal epicardial activations. Our algorithms used unit VCG orientations in the construction of transfer matrices, thereby designing the forward problem to be dynamic in nature.

The introduction of unit VCG priors in the statistical inverse problem not only addresses uncertainty issues but also identifies the orientations of reconstructed sources. The estimation of unit vectors of VCG is needed in order to understand and visualize the current flow directions that directly affect the MCG maps at the detector level. A slight deviation in the dipole orientation may lead to significant variations in the observation plane. The current study is compared with conventional methods, which used only static transfer matrices and a unit vector approach. The outcome of this research study helps medical doctors keep track of abnormal electrical pathways of the heart without performing invasive procedures.

## Supplementary Information

Supplementary Information.

## Data Availability

The datasets generated and/or analyzed in the current study are available from the corresponding author upon reasonable request.
