# Supplementary material for: Bayesian approach for localizing cardiac sources in Magnetocardiography using Vectorcardiography based total variational priors
Source: Sci Rep. 2025 Jul 11;15:25115. doi: 10.1038/s41598-025-09466-1 (PMC12254484; doi:10.1038/s41598-025-09466-1)
Supplement: Supplementary file 1 — Supplementary Information. [file 41598_2025_9466_MOESM1_ESM.pdf]

5 Appendix: Mathematical formulation and SCIRUN networks

The body surface potentials are computed using finite element method (FEM) before deriving the VCG vectors. This method constructs a stiffness matrix based on the geometries of heart and torso meshes and distributes the potentials inside each elemental meshes thereby covering the entire torso. After finding the potentials, electrodes are placed on the desired positions of the thorax and the corresponding ECG signals are recorded. A typical block diagram to obtain VCG from potential based FEM is shown in Figure 9.

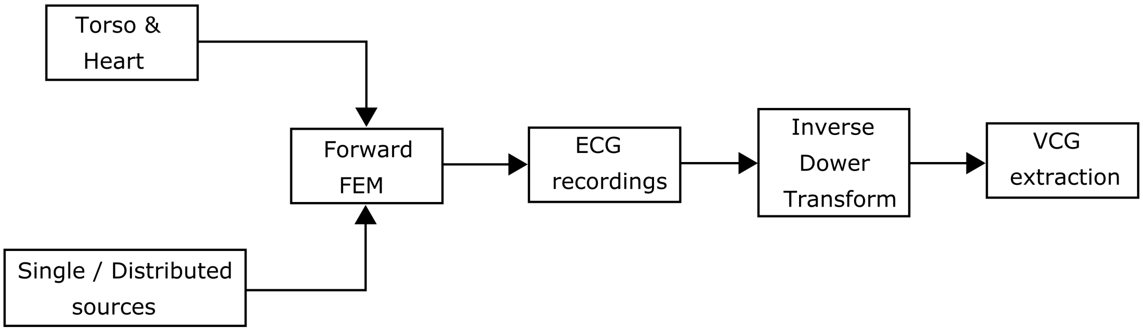

Figure 9. Procedure to extract VCG vectors from ECG signals

5.0.1 Forward FEM

The electric field induced by the heart source and the potentials are computed from the given volume conductor and an equivalent current dipole. The discretized heart and torso meshes are considered in the study to approximate potentials by solving the linear systems. These models contained tetrahedral finite elements with a conductivity tensor present in each element; indicating the flow of currents (electricity) through the region.[38]

According to the Maxwell's equation, the divergence of the electric field is equal to the charge enclosed inside the torso volume conductor.

Since no charge accumulation takes place inside the human body, the continuity equation is defined as the total current enters or leaves the volume is zero. The continuity equation is given by:

$$\nabla.\mathbf{J} = 0 \tag{40}$$

where $\mathbf{J}$ is the total current density. The electric field strength $\mathbf{E}$ is formulated as the gradient of the scalar potentials ϕ:

$$\mathbf{E} = -\nabla.\ \phi \tag{41}$$

The total current density is the sum of many source currents $\mathbf{J}_s$ and the conduction currents ($\sigma\mathbf{E}$).

$$\mathbf{J} = \mathbf{J}_s + \sigma\mathbf{E} \tag{42}$$

where σ is the conductivity of the tissues. Since the heart and torso volumes have no charge storage, the current flowing in and out of a volume or region becomes zero.

$$\nabla.\ (\mathbf{J}_s + \sigma\mathbf{E}) = 0 \tag{43}$$

by substituting equations eq. 41 and eq. 43, gives:

$$\nabla.\ \sigma\nabla\phi = \nabla.\mathbf{J}_s = -I_v \tag{44}$$

where $-I_v$ indicates the current source present in the region (or current per unit volume). This leads to the statement that the divergence of the electric field ($\nabla\phi$) is zero at the region with zero currents, that arrives to the generalized Laplacian equation for torso domain:

$$\nabla.\ \sigma\nabla\phi = 0 \tag{45}$$

First, the divergence of electric field ($\nabla.\ \mathbf{E}$) with stiffness matrix (A_{ij}) is evaluated by discretizing the Poisson's equations onto the tetrahedral regions of the torso volume.
The Laplace equation in terms of weighted residual form with scalar potentials is given by:

$$\int_{\Omega} \nabla.\ (\sigma \nabla \phi)\ w d\Omega = 0 \tag{46}$$

here Ω is the solution domain and w is the weights that yield in the Galerkin formulation while solving the linear systems. The boundary conditions are defined as:

$$\phi(x,y,z)|_{\Omega_{epi}} = V_{epi} \tag{47}$$

where Ω_{epi} is the epicardial surface domain and V_{epi} indicates the corresponding potentials at the nodes.
The finite element method is started by subdividing the torso geometry to a set of volume elements composed by vertices at a set of nodes. The potentials in the volume are then approximated by basis expansion:

$$\phi_V(x,y,z) = \sum_{i=1}^{Q} \phi_i N_i(x,y,z) \tag{48}$$

where ϕ_V represents the potentials in the volume, ϕ_i is the epicardial potentials at node i (unknowns in the inverse problem), N_i is the set of basis functions associated with each node Q in the discretized volume elements.
The potentials inside the torso volume is approximated from the known epicardial potentials by expansion of basis functions in eq. 50[38]. To solve the Laplace equation 47, the Galerkin method is employed by substituting equation 50 in equation 47 and both sides of the equations are multiplied by a set of test basis functions N_j and integrated over the domain volume:

$$\sum_{i=1}^{Q} \phi_i \int_{\Omega_{vol}} \sigma \nabla N_i\ \nabla N_j\ d\Omega_{vol} = 0 \tag{49}$$

where N_j is the basis functions associated with weighted residuals w expressed as:

$$w(x,y,z) = \sum_{j=1}^{Q} N_j(x,y,z) \tag{50}$$

The linear system equation 50 can be represented in the matrix form as:

$$A_{ij}\phi = 0 \tag{51}$$

where $A_{ij} = \int_{\Omega_{vol}} \sigma \nabla N_i\ \nabla N_j\ d\Omega_{vol}$ is the stiffness matrix, and ϕ is the unknown potentials. The stiffness matrix A_{ij} depends only on the geometry of torso and the choice of basis & test functions. The general conductivity profiles of skin layers are adapted from SCIRun dataset[39] are given in Table 5.

Table 5. Layers and conductivities of thorax volume[40]

Layers	Conductivity (S/m)
Air	0.0
Skin	0.00005
Fat	0.0000375
Muscle	0.000125
Lung	0.000054
Myocardium	0.000238
Heart blood	0.00068
Fat pad	0.00005
Bone	0.00068
Blood	0.00068
Heart	0.000238

The linear system $A_{ij}\phi = b$ was constructed to determine the scalar potentials ϕ through the field $\mathbf{E}$, where b is the current source representing the flux through nodes. The potentials are computed from the finite element simulation network with the help of SCIRun nets (Figure 10).

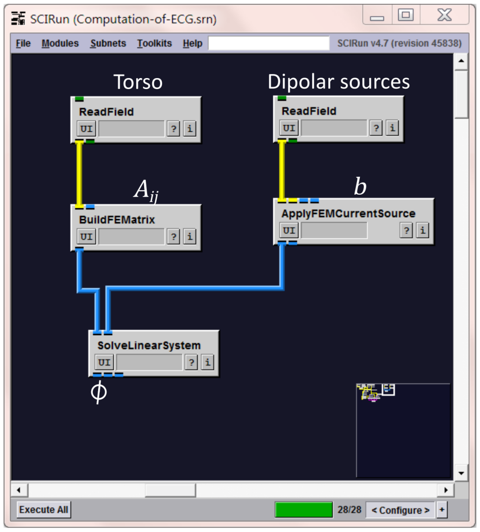

Figure 10. Forward FEM simulation nets in SCIRun framework

1. Open SCIRun → DataIO → ReadField → Torso & dipoles.
2. Go to SCIRun → Finite Elements → BuildFEMatrix is connected to ReadField of Torso.
3. Go to BioPSE → Forward → ApplyFEMCurrentSource module : connected with ReadField of dipole.
4. Go to SCIRun →Math → AddKnownsToLinearSystem module : connected with BuildFEMatrix and ReadMatrix containing known epicardial potentials.
5. SCIRun → Math → SolveLinearSystem : connected with the outputs of BuildFEMatrix and ApplyFEMCurrentSource modules.
6. Visualization →ShowField → SCIRun→ Render→ ViewScene.

The above algorithm explains the network connections setup to compute forward calculations using FEM for current sources inside the torso mesh volume. The steps 3 and 4 are modeled for single equivalent dipole and distributed sources respectively. The step 6 is used to visualize the calculated maps on the torso geometries in the view scene.

5.0.2 ECG recordings

In order to compute the potentials and record as ECG signals, similar SCIRun modules are connected as the previous one, but with a slight difference as epicardial maps are included in the prior information. These maps are associated with the heart socket using SetFieldData module and then passed to AddKnownsToLinearSystem module to make it as input current sources (b) for SolveLinearSystem. The other input to this module is connected from BuildFEMatrix of torso meshes which provides information of geometry and conductivity parameters as stiffness matrix A_{ij}. The first part of Figure 11a shows the network framed to compute the potentials throughout the tetrahedral elements present inside the torso mesh. The second part shows the network modules assembled to visualize amplitudes over time instants distributed on the heart, torso and electrodes. The detectors consisted of 300 body surface points spread over/ binding the thorax surface[36]. The electrodes are reduced to standard 12-lead precordials in the later stages to extract VCG. The construction of SCIRun nets to solve for linear systems include the following steps:

1. Load torso mesh, dipole sources/ epicardial potentials and electrodes.

2. Construct the finite element stiffness matrix A_{ij}.
3. The right hand side or the values of b is set to the strengths of dipolar sources or known values of epicardial potentials.
4. The unknowns inside the volume (ϕ) are determined by solving the linear system.
5. The approximated potentials reaching the thorax are recorded by placing the surface electrodes (Figure 11b).

The unipolar precordial/ chest leads (v1 to v6) are then selected from body surface electrodes based on approximate anatomical positions of the torso model and bipolar limb leads LI, LII and LIII are connected in the frontal plane of the model. The defined body surface electrode points set on the torso model record the resulting potentials at the points with respect to time as ECG signals.

The SCIRUN modules connected in the construction of forward problem (conventional method) as explained in the computational steps in section 1.2 is shown in figure 11

The computational steps described in 1.2.1 for construction of forward problem using VCG is demonstrated in figure 12. This is the extended version of the first computation.

Data availability

The datasets generated and/or analyzed in the current study are available from the corresponding author upon reasonable request.

Acknowledgment

The authors extend their gratitude to the Science and Engineering Research Board (SERB), Government of India, for financially supporting this research through the SERB SUPRA (Scientific and Useful Profound Research Advancement) scheme (grant number: SPR/2020/000423). Furthermore, the authors are grateful to the Manipal Institute of Technology (Manipal Academy of Higher Education), Manipal, India, for providing the necessary infrastructure for completing this work.

Author contributions statement

V.R.B designed the model and the computational framework. V.R.B carried out the implementation and performed the calculations. V.R.B, K.K and A.H analysed the results. V.R.B compiled the manuscript. All authors reviewed the manuscript.

References

1. Tilg, B. & Wach, P. Magnetic source imaging within the human heart from simulated and measured mcg data. In *Computers in Cardiology 1995*, 13–16 (IEEE, 1995).

2. Huiskamp, G. & Van Oosterom, A. The depolarization sequence of the human heart surface computed from measured body surface potentials. *IEEE Transactions on Biomed. Eng.* **35**, 1047–1058 (1988).

3. Hämäläinen, M. S. & Ilmoniemi, R. J. *Interpreting measured magnetic fields of the brain: estimates of current distributions* (Helsinki University of Technology, Department of Technical Physics, 1984).

4. Sarvas, J. Basic mathematical and electromagnetic concepts of the biomagnetic inverse problem. *Phys. medicine biology* **32**, 11 (1987).

5. Mariyappa, N. *et al.* Dipole location using squid based measurements: Application to magnetocardiography. *Phys. C: Supercond.* **477**, 15–19 (2012).

6. Burton, B. M. *et al.* A toolkit for forward/inverse problems in electrocardiography within the scirun problem solving environment. In *2011 Annual International Conference of the IEEE Engineering in Medicine and Biology Society*, 267–270 (IEEE, 2011).

7. Parker, S. G. & Johnson, C. R. Scirun: a scientific programming environment for computational steering. In *Supercomputing'95: Proceedings of the 1995 ACM/IEEE Conference on Supercomputing*, 52–52 (IEEE, 1995).

8. Malmivuo, J. & Plonsey, R. *Bioelectromagnetism: principles and applications of bioelectric and biomagnetic fields* (Oxford University Press, USA, 1995).

9. Ghosh, S. & Rudy, Y. Application of L1 norm regularization to epicardial potential solution of the inverse electrocardiography problem. *Annals biomedical engineering* **37**, 902–912 (2009).

10. Coll-Font, J. *et al.* New additions to the toolkit for forward/inverse problems in electrocardiography within the scirun problem solving environment. In *Computing in Cardiology 2014*, 213–216 (IEEE, 2014).

11. Zhukov, L., Weinstein, D. & Johnson, C. Independent component analysis for eeg source localization. *IEEE Eng. Medicine Biol. Mag.* **19**, 87–96 (2000).

12. Weinstein, D., Zhukov, L. & Johnson, C. Lead-field bases for electroencephalography source imaging. *Annals biomedical engineering* **28**, 1059–1065 (2000).

13. Van Uitert, R., Weinstein, D. & Johnson, C. Volume currents in forward and inverse magnetoencephalographic simulations using realistic head models. *Annals Biomed. Eng.* **31**, 21–31 (2003).

14. Jatoi, M. A., Kamel, N., Malik, A. S., Faye, I. & Begum, T. A survey of methods used for source localization using eeg signals. *Biomed. Signal Process. Control.* **11**, 42–52 (2014).

15. Mohammad-Djafari, A. From deterministic to probabilistic approaches to solve inverse problems. In *Bayesian Inference for Inverse Problems*, vol. 3459, 2–11 (International Society for Optics and Photonics, 1998).

16. Ouyang, H. *et al.* Non-probabilistic uncertain inverse problem method considering correlations for structural parameter identification. *Struct. Multidiscip. Optim.* **64**, 1327–1342 (2021).

17. MacKay, D. J. Bayesian interpolation. *Neural computation* **4**, 415–447 (1992).

18. Bishop, C. M. *Pattern recognition and machine learning* (springer, 2006).

19. Arinbjarnarson, T. M. *Bayesian approach to the ill-posed eeg inverse problem.* Ph.D. thesis, Ph. D. dissertation, Technical University of Denmark (2007).

20. France, J. J., Gur, Y., Kirby, R. M. & Johnson, C. R. A bayesian approach to quantifying uncertainty in tikhonov solutions for the inverse problem of electrocardiography. In *Computing in Cardiology 2014*, 529–532 (IEEE, 2014).

21. Babacan, S. D., Molina, R. & Katsaggelos, A. K. Bayesian compressive sensing using laplace priors. *IEEE Transactions on Image Process.* **19**, 53–63 (2009).

22. Babacan, S. D., Molina, R. & Katsaggelos, A. K. Fast bayesian compressive sensing using laplace priors. In *2009 IEEE International Conference on Acoustics, Speech and Signal Processing*, 2873–2876 (IEEE, 2009).

23. Lee, J. & Kitanidis, P. Bayesian inversion with total variation prior for discrete geologic structure identification. *Water Resour. Res.* **49**, 7658–7669 (2013).

24. Bardsley, J. M. Laplace-distributed increments, the laplace prior, and edge-preserving regularization. (2012).

25. Järvenpää, M. & Piché, R. Bayesian hierarchical model of total variation regularisation for image deblurring. *arXiv preprint arXiv:1412.4384* (2014).

26. Babacan, S. D., Molina, R. & Katsaggelos, A. K. Parameter estimation in tv image restoration using variational distribution approximation. *IEEE transactions on image processing* **17**, 326–339 (2008).

27. Babacan, S. D., Molina, R. & Katsaggelos, A. K. Total variation image restoration and parameter estimation using variational posterior distribution approximation. In *2007 IEEE International Conference on Image Processing*, vol. 1, I–97 (IEEE, 2007).

28. López, A., Cortés, J. M., López-Oiler, D., Molina, R. & Katsaggelos, A. K. Hyperparameters estimation for the bayesian localization of the eeg sources with tv priors. In *2012 Proceedings of the 20th European Signal Processing Conference (EUSIPCO)*, 489–493 (IEEE, 2012).

29. Bhat, V. R. & Anitha, H. Computational imaging of the cardiac activities using magnetocardiography. *J. Med. Eng. & Technol.* **43**, 401–410 (2019).

30. Hall, J. Guyton and hall textbook of medical physiology: Enhanced e-book. 2010.

31. Vozda, M. & Cerny, M. Methods for derivation of orthogonal leads from 12-lead electrocardiogram: A review. *Biomed. signal processing control* **19**, 23–34 (2015).

32. Edenbrandt, L. & Pahlm, O. Vectorcardiogram synthesized from a 12-lead ecg: superiority of the inverse dower matrix. *J. electrocardiology* **21**, 361–367 (1988).

33. Feher, J. J. *Quantitative human physiology: an introduction* (Academic press, 2017).

34. Andra, W. & Nowak, H. *Magnetism in medicine: a handbook* (Wiley Online Library, 1998).

35. MacLeod, R. S. *et al.* Scirun/biopse: Integrated problem solving environment for bioelectric field problems and visualization. In *Biomedical Imaging: Nano to Macro, 2004. IEEE International Symposium on*, 640–643 (IEEE, 2004).

36. Van Oosterom, A. & Oostendorp, T. Ecgsim: an interactive tool for studying the genesis of qrst waveforms. *Heart* **90**, 165–168 (2004).

37. Van Oosterom, A., Oostendorp, T., Huiskamp, G. & Ter Brake, H. The magnetocardiogram as derived from electrocardiographic data. *Circ. research* **67**, 1503–1509 (1990).

38. Andrew J Pullan, L. K. C. & Buist, M. L. *Mathematical modelling the electrical activity of the heart : from cell to body surface and back again* (World Scientific, New Jersey, 2005).

39. MacLeod, R. S. *et al.* Scirun/biopse: Integrated problem solving environment for bioelectric field problems and visualization. In *Biomedical Imaging: Nano to Macro, 2004. IEEE International Symposium on*, 640–643 (IEEE, 2004).

40. Burton, B. M. *et al.* A toolkit for forward/inverse problems in electrocardiography within the scirun problem solving environment. In *2011 Annual International Conference of the IEEE Engineering in Medicine and Biology Society*, 267–270 (IEEE, 2011).

41. ECGSIM. Magnetocardiography. *ecgsim.org*. https://www.ecgsim.org/downloads/other13/help/mcg.html (accessed 21 Apr 2025).

42. V. R. Bhat and [Co-author(s)], "Reconstruction of cardiac activities from Vectorcardiography and Magnetocardiography using Bayesian approach with coherence mapping," in *Proceedings of [Conference Name]*, 2023, pp. xx–yy.

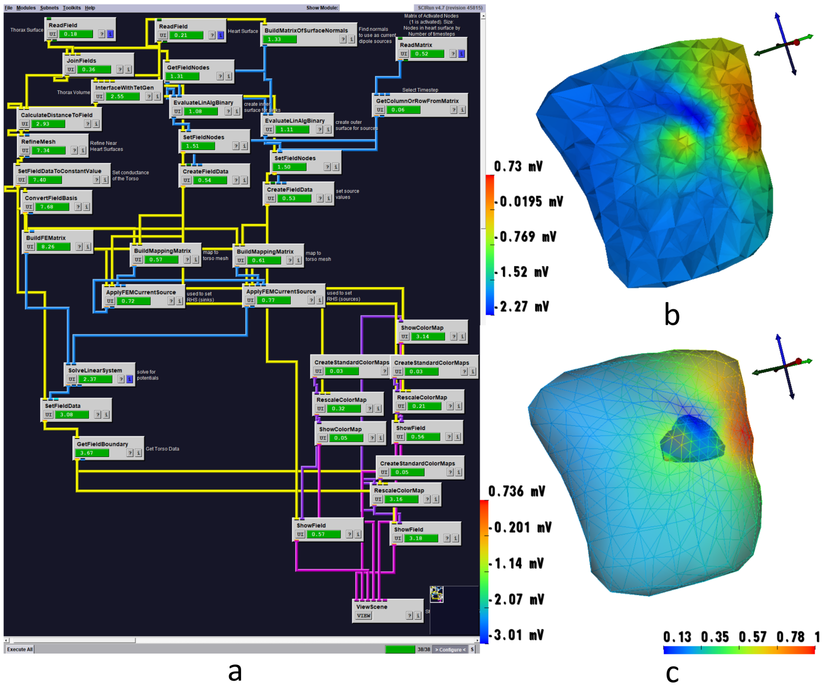

Figure 11. Potential simulation nets in SCIRun framework: a. Computation of body surface potentials from epicardial maps and modules connected to visualize the distributed potentials, b. and c. Computed maps collected at surface along with heart and torso meshes visualized in ViewScene of SCIRun during the time instant $t = 200$ ms. Colormaps indicate the maximum (red) and minimum (blue) amplitudes of potentials. Bottom: colormap of heart potentials, Left: Torso potentials colormap

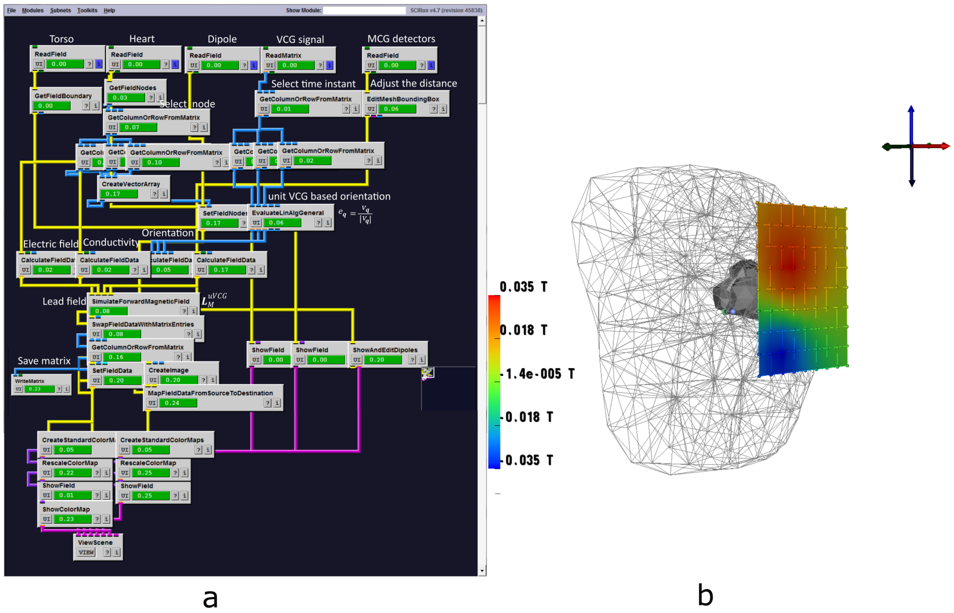

Figure 12. Lead field based on unit VCG constructed in SCIRun environment: a. Dynamic lead field $\mathbf{L}_M^{uVCG}$ is computed for all nodes separated by atrium Q_a and ventricular nodes Q_v in desired time instants uP, $uQRS$ & uT and saved using WriteMatrix module, b. Generation of lead field at the detectors due to a dipole assigned with unit VCG of R instant.
